# Professor W. Stone’s Treatment of Yellow Fever

**Published:** 1856-02

**Authors:** 


*Professor W. Stone's Treatment of Yellow Fever.* — We extract from the New York Medical Tines the following remarks of Prof. Stone, whose acknowledged ability and success in the treatment of yellow fever entitle his testimony to great weight. The learned gentleman's views were delivered at a recent meeting of the New York Academy of Medicine.

*Treatment.*—Yellow fever is a self-limited disease; it is not to be treated —it is to be managed. All that is to be done, is to keep the patient alive for a certain time, and he will get well.

The disease is ushered in with a chill or slight rigor, often scarcely noticeable, followed by heat in the forehead, pain in head, limbs and back. This is again followed by a hot fever, and if the patient be kept under cover, and carefully treated, these symptoms will quietly terminate in two or three days; but if left to themselves to toss about and not remain under cover, the sweating stage passes on for five or six days, and collapse, black vomit, and death result.

The only treatment found by Dr. S. to be useful, is to favor the efforts of nature in prolonged sweating, calm, and rest of the system, and the fever will generally be got rid of in the first two or three days. It terminates in favorable cases on or before the third day, leaving the skin natural. Those who treat it otherwise than expectantly, do not understand the nature of the disease.

Among those who may be said to understand the disease, there are two methods of treatment: the expectant—cups to temples to relieve cephalalgia, slight laxatives to open the bowels, hot baths under the bedclothes. Others give quinine. Dr. S. was the first to do so, whoever has the credit, but no matter about that. The only difference is that they do not give it with any specific object. His method was a full dose at the beginning of the disease, but not afterward. Thus given, it promoted and prolonged the sweating stage, and while this was kept up, the patient was safe. It was remarked, that they would get well without quinine, where it was generally prescribed. He was physician for many years to a hospital where there were 40 to 50 cases a day, and he noted that those in favor of this quinine treatment were about 10 per cent.

Dr. S. remarked, that in 1847 he treated forty cases in succession by quinine, among mechanics, who had no nursing except what was provided by friends, and did not lose a single patient. It was in a favorable epidemic, but considering their destitution of proper nursing, deaths would have been as likely to occur as in a worse epidemic.

As to the use of *calomel* in this disease, there is no possible condition of the system where there could be any benefit derived from its use — there was no local disease. The liver has nothing to do with it. He knew this, for he had followed the patients of the Calomelites to the dead house in plenty. While serving as surgeon to the Charity Hospital, the medical side of the hospital was full, and there were several mechanics who applied for admittance, and wished to be treated by Dr. S., and as he had some empty beds, he received them. But one was sent to the medical wards, and he gave him foot-baths, kept him warm, regulated his drinks, &c., and gave quinine. The attending physician came in the morning; he ordered a drachm of calomel, but Dr. S. put up magnesia. The next day he was better, and the doctor repeated the dose, but he took the liberty to repeat the magnesia. He got well, and thenceforward was one of the attending physician's "brag cases," for he dared not tell him of his doings. Then came new physicians from Paris, full of Broussais' theory, and they bled and boasted of their successes also for a while, and in truth they did succeed as well as the former. Then came eclectics with fanciful theories; they gave a little calomel to disgorge the liver a little, cups a little, leeches a little, and with a result very little different from the others. In fact some will get well in spite of the treatment, and then again some fevers are fatal in the beginning. When, however, they are brought side by side, and we can observe them *en masse*, we can more properly judge of the relative value of the different modes of treatment, than in the isolated cases met with in ordinary practice. The difference is very manifest—all perturbating treatment is alike bad.

There are some peculiarities in the disease that might not at first strike one—the disturbed nervous system, and especially delirium—one of the worst symptoms. This may appear at first, but not usually. Its first evidence is restlessness and want of sleep; objects are seen very much as in *mania-a-potu*. Narcotics produce stupor and death, for the patients with this disease are peculiarly susceptible to morphine: stimulants are much better. You must watch to give the stimulants as early as possible; they then sweat off, and are relieved in 24 to 36 hours; but even then they must not be disturbed—if raised up, they faint away. Perfect and absolute rest, body and mind, are indispensable. If they are excited, the heat returns, and they die. Watch for sleeplessness, and give minute anodynes and stimulants, such as they are able to bear. Of those cases that run on and approach the period of black vomit, if managed properly as to drinks, by avoiding bitter infusions, &c., very many recover; and those that do not vomit would have the black vomit if they vomit at all. Some have a preference for certain kinds of drink, as porter, others prefer brandy, &c. Give those agreeable to the palate. As they approach the black-vomit period with previous restlessness and acid secretions, give some alkali, with minute doses (say a 20th or a 30th of a grain) of morphine, with champagne, ale, beef-essence, &c. Impart to the patient a feeling of safety and security. And yet I have thought, in proportion to the mildness of the disease was the danger; for quiet is absolutely necessary, and coercion does not answer. The patient is to be managed, not treated.

Footh-baths under the clothes, will often produce favorable sweats. When in a state of dry heat, forced perspiration is bad; sponging with tepid water is then better. The douche is but of temporary benefit, and the subsequent reaction leaves the patient worse. Sponging with lemon-juice, sweet oil, and salt is used by the Creoles and Spaniards, but pure water is better. All that is to be done is to ease them through.

Dr. Gardner stated that Dr. Ashbel Smith laid great stress on covering, and said the patient should be enveloped in blankets and carefully excluded from the air. Was this correct, and what drinks are proper?

Dr. Stone. — Careful covering of the entire body and limbs is absolutely requisite, but not to swelter under too much covering. If the hands were but exposed, sometimes the heat would return and a relapse ensue. Some mild diaphoretics may be given; such drinks as the patients desire; one year all want brandy and water, other years malt liquors. Give that which is desired, and carefully avoid even the nervous shock caused by a bitter or disagreeable medicine. Sponging the body under the clothes, ice water to head, generally were followed by reaction and more pain. Dr. Cartwright had pursued the opposite plan of enveloping the head in warm fomentations.

Dr. Corson wished to know the doses of quinine which Dr. S. gave, and the vehicle.

Dr. Stone replied, that he usually gave a single dose of fifteen or twenty grains, according to circumstances, at the onset, perhaps ten grains more twelve hours after, but none unless on the first day; and the second day it is entirely useless, and after that actually injurious, although they bear it better than any other remedy. It causes vomiting when given late, and is not necessary, for its effects last several hours after its administration.

